# Healthcare analytics—A literature review and proposed research agenda

**DOI:** 10.3389/fdata.2023.1277976

**Published:** 2023-10-05

**Authors:** Rawan Elragal, Ahmed Elragal, Abdolrasoul Habibipour

**Affiliations:** Department of Computer Science, Electrical, and Space Engineering, Luleå University of Technology, Luleå, Sweden

**Keywords:** healthcare, data science, data analytics, AI, big data, machine learning, literature review

## Abstract

This research addresses the demanding need for research in healthcare analytics, by explaining how previous studies have used big data, AI, and machine learning to identify, address, or solve healthcare problems. Healthcare science methods are combined with contemporary data science techniques to examine the literature, identify research gaps, and propose a research agenda for researchers, academic institutions, and governmental healthcare organizations. The study contributes to the body of literature by providing a state-of-the-art review of healthcare analytics as well as proposing a research agenda to advance the knowledge in this area. The results of this research can be beneficial for both healthcare science and data science researchers as well as practitioners in the field.

## 1. Introduction

Today, several aspects of human lives, such as social, economic, and cultural, are intertwined with the rapid growth of new technologies. This has significantly influenced various sectors and industries, including healthcare (Von Lubitz and Wickramasinghe, 2006). Examples of these trends include, but are not limited to, using Internet of Things (IoT) sensors and wearable devices, telemedicine, cloud computing, Artificial Intelligence (AI)-based healthcare solutions, and so forth. Data science (DS) is becoming increasingly popular in a wide range of real-world applications and services. It employs multiple methodologies on a wide range of data for several objectives. Data analytics (DA), data mining, databases, high-performance computing, cloud computing, machine learning (ML), mathematical and statistical modeling, and visualization are examples of the tools and technologies used in DS. The aforementioned DS techniques can be applied to various datasets, e.g., biodiversity, census, diseases and healthcare, environment, genomics, social networks, transportation, and the web, for visualization, analysis, and interpretation (Leung et al., 2021). Most of these technological advances rely on vast amounts of data, the so-called big data (BD). Using BD has its own challenges and complexities, particularly in the healthcare industry, in which the process of DA is even more sensitive as it relates to humans' health and lives (Gupta et al., 2013). Despite this, big data analytics (BDA) has tremendously contributed to the healthcare industry, not only for identifying patients' health problems but also for the treatment of various diseases by using smart technologies and solutions. Moreover, BDA has numerous implications and benefits, including cost reduction, increased potential for tailoring personalized medicine, use in large-scale genetic studies, benefits for public health, improved patient-provider relationships, and an increased likelihood of drug discovery (Wang and Alexander, 2020; Awrahman et al., 2022).

While numerous studies have explored the intersection of healthcare science and data analytics, there is a notable scarcity of comprehensive research that addresses how and to what extent big data analytics (BDA) can assist the healthcare sector in addressing contemporary challenges where data science can play a role in identification and resolution (Awrahman et al., 2022). Accordingly, this paper aims to dig deeper into the application of BD and DA algorithms to identify and address healthcare concerns. In so doing, the paper is guided by the following research question:

- *How could the use of data science elements (big data, and analytics algorithms) help identify, address, or contribute to the solution of some of the healthcare challenges and concerns?*

The paper is structured as follows: Section 2 describes the overall methodology that has been used to review the literature. Section 3 provides the necessary theoretical background knowledge for the paper in DS, BD, and analytics. Section 4 describes the nature of the healthcare system. Section 5 reviews the research conducted in healthcare analytics. Then comes Section 6, which identifies research gaps. Section 7 provides a proposed future research agenda, and finally Section 8 introduces the conclusion of the paper.

## 2. Method

To address the aim of this research, we followed a concept-centric literature review approach as outlined by Webster and Watson (2002). This approach contrasts with the author's centric approach, in which the readers are usually familiar with the main topic and there are already studies available that discuss the main topic in detail. We chose the concept-centric method since it allows us to systematically synthesize the literature and enables us to make an initial classification of the healthcare analytics literature. In the preliminary stage of the literature review, a careful manual search was conducted across the core academic journals in the field. of healthcare analytics. This exploratory search resulted in 13 articles. We then went through the contents of each of these journals and conferences and manually looked for the relevant articles by reviewing titles, abstracts, and keywords.

The next phase involved deploying a carefully curated combination of keywords to execute searches in a set of widely recognized databases including Scopus, Web of Science, EBSCO, PubMed, and MEDLINE, using the “search terms” for literature searches. The keywords that were used for this literature review were: healthcare, analytics, big data, AI, and IoT. Any meaningful combinations of these keywords were included as a “search term.” Examples of the search terms used were healthcare analytics, big data for healthcare, AI and healthcare, AI in healthcare, and IoT in healthcare analytics. Other search techniques were also applied, including logical combinations of the keywords, such as “healthcare” AND “IoT” AND “analytics”; “big data” AND “healthcare”; “healthcare” AND “AI”; and so forth. This careful methodology resulted in the compilation of a list of 211 potential articles. Subsequently, we conducted an evaluative phase in which each article underwent screening based on its title and abstract to assess its relevance to the research question. Following this thorough examination, 15 articles were deemed pertinent and subsequently included in the literature review.

**Figure 1:**
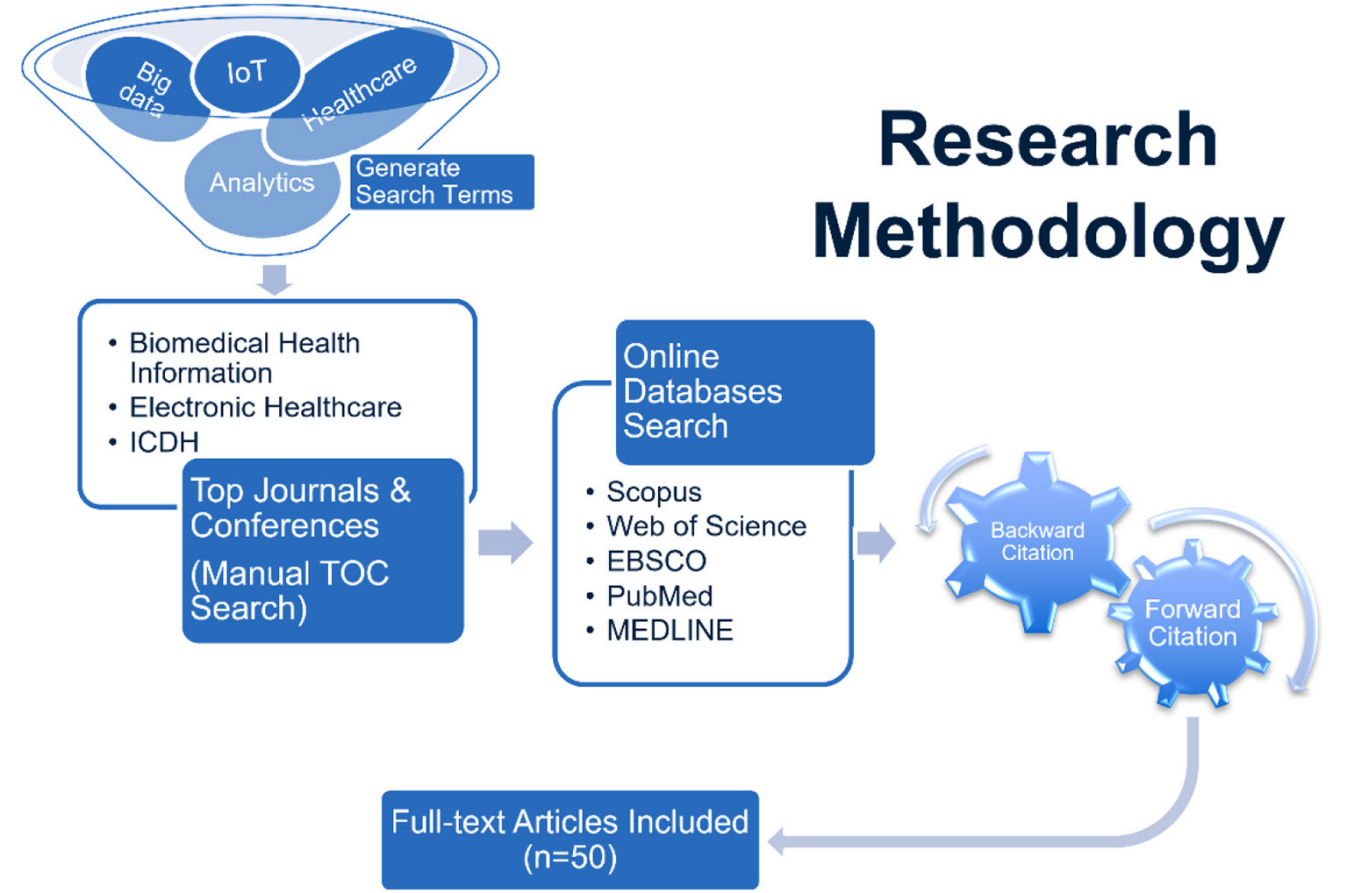
Research methodology.

Finally, further relevant studies were identified through backward and forward citation analysis based on Webster and Watson (2002) recommendation. This approach was employed because the number of relevant findings in the previous steps was too small to obtain reliable results. The citation tracking process added an extra 22 articles to our literature collection. In total, we reviewed 50 articles, with each one playing a role in shaping our research findings and conclusions. Only English literature was considered in this review, and due to the emerging nature of healthcare analytics, no time limitation was set for this review. Figure 1 summarizes the research methodology followed in this literature review.

## 3. Theoretical background

**Figure 2:**
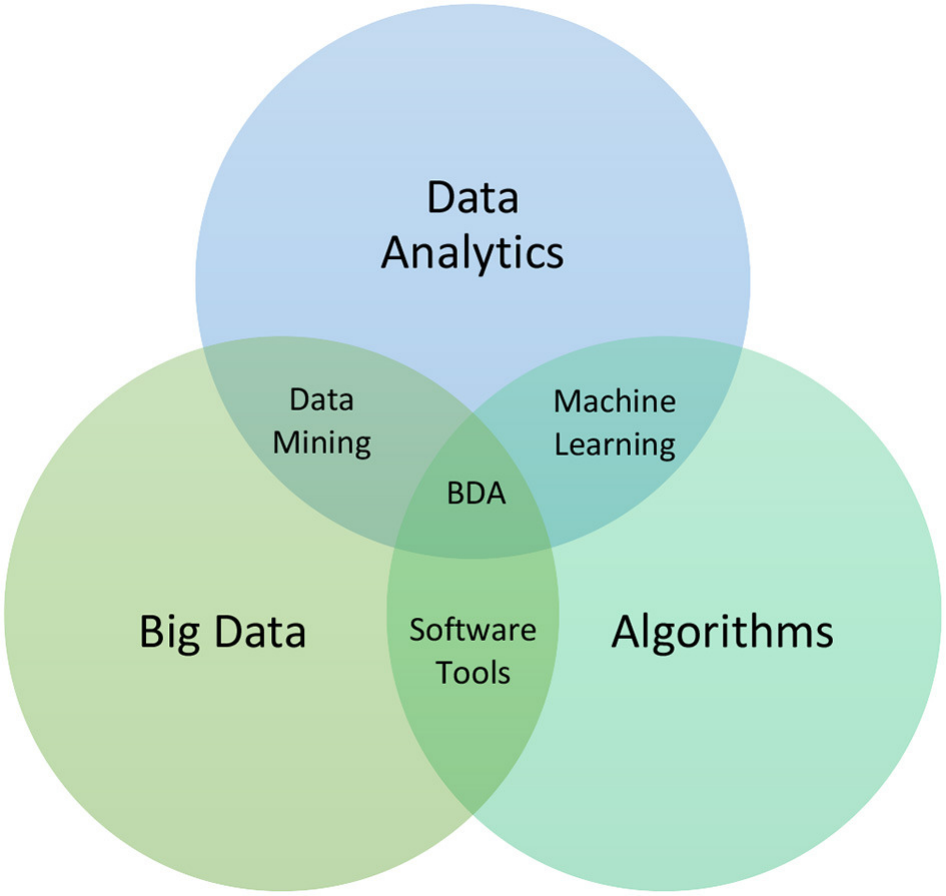
Data science subsets.

Many disciplines nowadays seek to use DS, which involves the use of algorithms running on top of BD following a process, to extract knowledge and insights from structured and unstructured data and henceforth be able to address classical problems, e.g., healthcare, via innovative technologies. In this section, we provide theoretical background on BD, DS, AI, ML, and IoT to use them later in the paper as a lens through which to review the recent developments in healthcare that have resulted in what is known as healthcare analytics. Analytics is a multidisciplinary field that draws on a wide range of skills and knowledge from multiple areas, including BD, DS, AI, ML, and IoT, which collectively work in a contextual domain of expertise. One of the key aspects of analytics is the ability to work with and analyze various types of data, including structured, unstructured, and streaming data (Elgendy et al., 2021). Analytics has penetrated several areas of our disciplines, enabling it to be at the forefront of current developments in society to enable digitalization, in food to enable food analytics, in healthcare to enable digital healthcare, etc. In Figure 2, you will find a representation of some of these areas. In the following sections, we provide theoretical background on the ingredients of the analytics domain.

### 3.1. Big data

Nowadays, it is hard to open a popular publication, online or in print, without encountering a reference to DS, analytics, BD, or some mix thereof (Agarwal and Dhar, 2014). BD is data of such volume, spread, diversity, and velocity that it necessitates the employment of technical structures, analytics, and tools to derive insights that uncover hidden information and create value for businesses. BD is defined by three key features: volume, velocity, and variety (aka the three Vs). Volume is the primary attribute of BD and refers to its size and enormity (Lau et al., 2016). Velocity is the rate at which data is changing or being created. Variety includes the different formats and forms of data gathered from structured and unstructured sources. BD can be measured by size in TBs or PBs, besides the number of tables, records, files, or transactions. Moreover, one of the things that makes BD actually big is that it comes from more sources than ever before, including IoT data, clickstreams, logs, and social media. When these sources are used for analytics, typical structured data is joined by unstructured data such as text and human language, as well as semi-structured data such as JSON, extensible markup language (XML), or rich site summary (RSS) feeds. Furthermore, multidimensional data from a data warehouse can be retrieved to provide historical context for BD. Thus, variety is equally as crucial as volume in BD. Streaming data, which is acquired in real-time from websites, is at the cutting edge of BD. Some researchers and organizations have proposed adding a fourth V, veracity. The quality of data is the emphasis of veracity. This characterizes BD quality as good, bad, or undefined due to data incompleteness, inconsistency, latency, ambiguity, deception, and approximations (Elgendy and Elragal, 2014).

Actually, not just firms and governments generate data; we are all data generators now (McAfee and Brynjolfsson, 2012). We generate data using mobile phones, social network connections, GPS, and so on. However, most of such data is not structured in such a way that it can be stored and/or processed in a standard database management system (DBMS). This necessitates the use of BDA approaches to make sense of such data.

**Figure 3:**
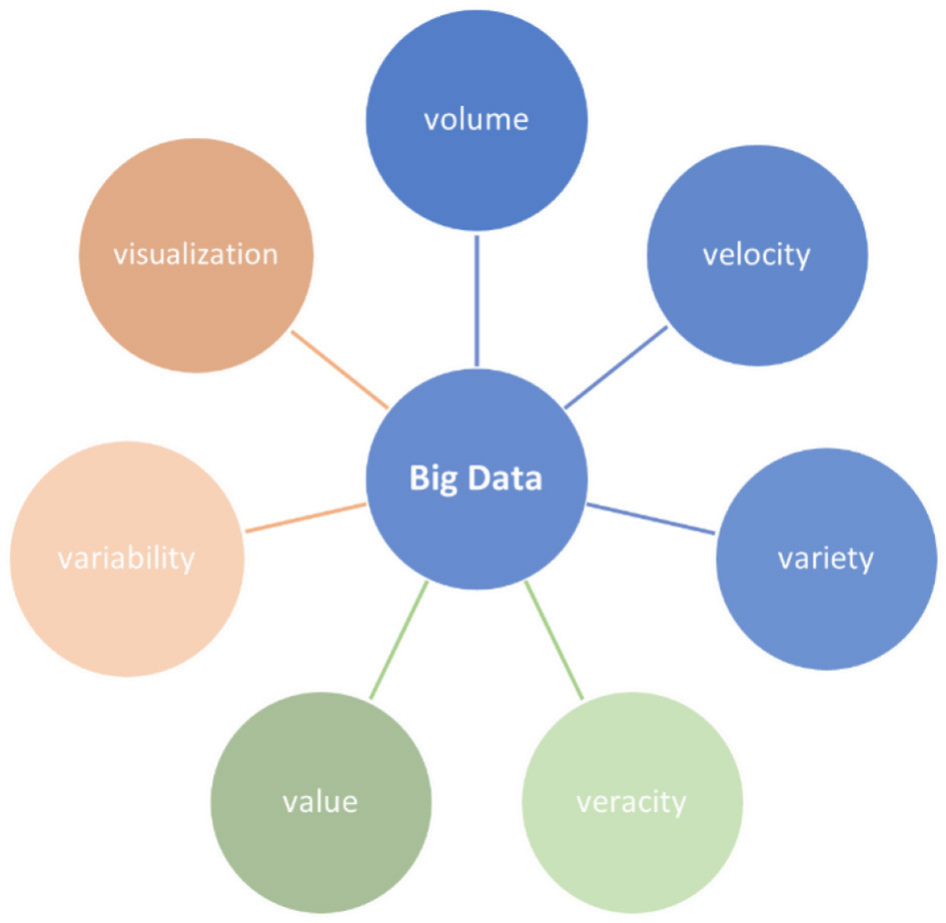
Big data Vs.

Due to its high volume, velocity, variety, veracity, and value, BD cannot be managed using traditional tools and techniques (Elgendy and Elragal, 2014). Where value points to the strategic and informative benefits of BD and veracity pertains to the data sources' reliability. Variability and visualization have also been incorporated recently. Figure 3 represents the BD Vs discussed in this paper; the ones in blue are the main three.

By making more types of information available and useful at a higher frequency, BD may unlock enormous value by improving product and service development, enhancing performance, optimizing decision-making (DM), and leading to improved and more up-to-date management decisions (Manyika et al., 2011). To collect, analyze, link, and compare such datasets, however, appropriate technology, processing capacity, and algorithmic precision are required. For so, BD can provide a greater level of intellect and knowledge that can provide previously inconceivable discoveries with the truth, objectivity, and accuracy (Boyd and Crawford, 2012). Howbeit, simple ownership of BD is insufficient to generate a sustained competitive advantage, which requires the ability to compile structured and unstructured data, analyze massive amounts of such data, and use the insights to inform decisions (Amankwah-Amoah and Adomako, 2019). Accordingly, it is well recognized in literature that BD distinguishes from traditional data and necessitates new methods for storage, management, and processing than previous data and information (Elgendy et al., 2021).

### 3.2. Data science

DS is the systematic extraction of non-obvious useful patterns and knowledge from data (Dhar, 2013; Kelleher and Tierney, 2018). It aims to advance research, support organizational DM, and enable a data-driven society (Ahalt, 2013). The definition shows that DS shares similarities with science, scientific methods, and business analytics. Unsurprisingly, DS shares a lot of the definition of science and scientific method, that is, “*principles and procedures for the systematic pursuit of knowledge involving the recognition and formulation of a problem, the collection of data through observation and experiment, and the formulation and testing of hypotheses*” (Merriam-Webster, 2022). In the DS domain and similar domains (e.g., decision support systems), “knowledge” refers to insights and claims that can be made from factual data analysis (Rizk and Elragal, 2020). DS is predicated on the reality that massive data is now widely accessible and that analytical algorithms used to reveal insights, beneficial patterns, and hidden information from data are now readily available in the form of tools and libraries, e.g., Python libraries.

### 3.3. Artificial intelligence and machine learning

The use of AI in our daily lives is increasing exponentially. As per Luckin et al., “*AI is a computer system that has been designed to interact with the world through capabilities (e.g., visual perception and speech recognition) and intelligent behaviors (e.g., assessing the available information and then taking the most sensible action to achieve a stated goal) that we would think of as essentially human”* (Luckin et al., 2016). Another definition of AI states that it is an area of science aimed at assisting machines in uncovering more human-like answers to challenging problems. This basically entails taking traits from human intellect and applying them to algorithms. Despite its close link with computer science, AI has links with a wide range of other key disciplines, including mathematics, biology, psychology, cognition, and philosophy (Tirgul and Naik, 2016).

Perhaps the most intriguing definition was the slogan for Thinking Machines, Inc., a now-defunct computer company: “*making machines that will be proud of us*” (Murphy, 2000). AI is a controversial term that sparks ongoing philosophical discussions about whether a machine can ever be intelligent. As a result, to escape the debate surrounding the name AI, many researchers describe their work as “intelligent systems” or “knowledge-based systems” (Murphy, 2000). Each day, new AI applications, spanning from ML to robotics and other AI-enabled technology, penetrate our lives.

At the heart of AI is ML, which is a collection of algorithms used to analyze datasets. ML aims to process and analyze large datasets by employing advanced analytics algorithms. It enables the development of practical ideas for assessing performance, establishing competitive advantages, and functioning as a new productive, innovative, and improved data-driven DM platform (Wamba et al., 2016). Moreover, analytics includes the technology, processes, tools, and techniques, or analytical approaches, that may be employed on datasets to produce valuable insights and actionable descriptive, prescriptive, and predictive results (Mikalef et al., 2018). The analytics engine, at its core, employs a series of complex algorithms to discover hidden patterns in large datasets (Russom, 2011). The top 10 data mining algorithms were determined during the IEEE 2006 International Conference on Data Mining (ICDM) based on expert nominations, citation counts, and a community survey. In sequence, those algorithms are: C4.5, k-means, support vector machine (SVM), Apriori, expectation maximization (EM), PageRank, AdaBoost, k-nearest neighbors (kNN), Naïve Bayes, and CART. Classification, clustering, regression, association analysis, and network analysis are all covered (Elragal and Klischewski, 2017).

### 3.4. Internet of things

In 1998, Kevin Ashton first introduced the term “IoT.” Things in IoT are any existing objects that are either communicating or non-communicating. The architecture of IoT is considered a three-layer technology. These three layers are the perception layer, the network layer, and the application layer. Machine-to-Machine (M2M) communications are the basis of IoT. Without intervention from humans, the IoT communicates between two machines (Aazam et al., 2014).

IoT is an ecosystem integrating physical objects, software, and hardware to allow them to interact with each other. It is a sophisticated network of uniquely identifiable “things,” where each of these objects connects to a server that provides efficient and suitable services. They communicate with each other and with the physical world by transferring pertinent data from both the physical and virtual worlds. These things can respond autonomously to the surrounding world events. All these processes can activate some actions and create services through human intervention or M2M communication (Ahmadi et al., 2019; Hussain et al., 2022).

IoT has application in several areas, such as connected industry 4.0, smart cities, smart homes, smart energy, self-driving cars, smart agriculture, and healthcare. Since the healthcare sector is continually looking for innovative methods to deliver services while lowering costs and improving quality, its reliance on IoT technology will continue to grow. The adoption of such technologies enables patients to practice self-care principles, resulting in cost-effective healthcare services, higher patient satisfaction, and better self-management.

## 4. The healthcare system

In this section, the main modules constituting the current healthcare system will be highlighted. Firstly, records, such as health, financial, administrative, patient demographics, practitioners', etc. These are the foundations for building patients' profiles, designing treatment plans, and so on. Then there is data, without which we will not have records or even an established firm. Additionally, diagnosis and biomarkers are the keys to curing any patient, and they either make or break the efficacy of the treatment and/or recovery plan. Moreover, genetic variations and gene libraries could help practitioners take a whole other direction when it comes to treatment plans or adjusting lifestyle habits for patients, which brings us to a vital and emerging field known as precision medicine. Further, patient compliance is something crucial to bear in mind when dealing with patients; convenience plays a huge role in following prescriptions. Finally, in social forecasting, insights from social media could help trace or interpret a bunch of unwanted social behaviors or disease outbreaks; hence, they could help us prevent or, at least, prepare for what is ahead.

In the next subsection, we will state the reality behind generating and storing health records in the healthcare system.

### 4.1. Electronic health records

The economic situation has resulted in medical field facilities and health care professionals being heavily dependent on paid services, thus hindering the progress of technology in these areas. Technological advancements have resulted in an avalanche of medical data from numerous sectors. Yet, the data collected from various sources is unstructured, noisy, and poorly annotated; hence, it is not completely utilized to generate relevant insights for therapeutic applications (Wang and Alexander, 2019).

However, the Health Information Technology for Economic and Clinical Health (HITECH) Act of 2009 encouraged hospitals to use electronic health records (EHR). Patient demographics, clinical notes, prescriptions, procedures, lab test results, diagnosis, and so on are all stored in the hospital's EHR. EHR data can aid in therapy selection, discovering patient similarities, integrating genetic data for individualized treatment, forecasting hospital length of stay, and predicting patients' readmission chances. However, because of the high heterogeneity, a high chance of missing or erroneous entries is possible, resulting in practitioners' reluctance to use these technologies, primarily because they still require abductive reasoning to acquire clinical insights from them to execute effective diagnosis. Even though hospitals have successfully used EHR for various administrative and corporate chores such as patient logging, asset and transfer management, and mostly financial transactions, there is still a need to identify methods to properly use EHR for patient diagnosis. Nevertheless, several difficult integration challenges, as well as labeled data scarcity for training models and privacy concerns, impede the successful use of these systems to produce effective care (Harerimana et al., 2019). In the following sub-section, you will get introduced to some data collection tools, such as biosensors.

### 4.2. Data collection and wearable biosensors

Nowadays, our healthcare services are more expensive than earlier, and most patients are compelled to stay in hospitals for their treatment period. These difficulties can be overcome by using IoT technologies, where patients are remotely monitored. The World Health Organization (WHO) developed a list of characteristics that make an appropriate diagnosis test for resource-limited sites: ASSURED, which stands for affordable; sensitive; specific; user-friendly; rapid treatment and robust use; equipment-free; and finally, delivered to people in need (WHO, 2006). Wearables (aka wearable biosensors) have been part of a larger multidisciplinary healthcare initiative to employ mHealth to improve data collection, diagnosis, treatment, and health insights. Despite differences in definition, the WHO's Global Observatory for eHealth described mHealth as “medical and public health practice supported by mobile devices, such as mobile phones, patient-monitoring devices, personal digital assistants, and other wireless devices” (Witt et al., 2019). Biomedical sensors collect and convert biomedical signal variables into electrical impulses to interpret raw physiological characteristics into meaningful digital health information. A biomedical sensor connects a biological and an electronic system. Biomedical sensors can be physical or chemical, according to scientific classification (gas, electrochemical, photometric, or bioanalytic). Physical sensors evaluate physical quantities (body temperature, blood flow, blood pressure, muscle displacement, bone growth, and skin moisture), whereas chemical sensors examine chemical substance concentrations (Aileni et al., 2015).

IoT technologies collect and share real-time health data from patients to healthcare providers, lower healthcare services cost, and allow for rapid treatment of health concerns before being critical. According to the WHO's study on aging and disability, people's life expectancy has increased and people who are older are more vulnerable to chronic diseases, impairments, and hospitalizations (Marengoni et al., 2011). Soon, healthcare delivery will shift from hospital-to-home balance to homecare services. Home monitoring is one of the most impressive uses of Wireless Sensor Networks (WSN), which utilizes heterogeneous sensors to detect human activity. Incorporating various IoT components into home care and medical systems is increasing in popularity, particularly for events like fall and seizure detection. Caregivers can thus provide better care and take prompt actions to avoid potentially harmful situations during seizures. In the below sub-section, several diagnostic and biomarker detection techniques will be introduced.

### 4.3. Diagnosis and biomarker detection

As a disease progresses in a person, changes in their physiological status occur in response to disease progression. A biomarker (e.g., mRNA expression patterns, circulating DNA and tumor cells, proteins, proteomic patterns, lipids, metabolites, imaging modalities, or electrical signals) is a property that can be quantified and assessed to indicate biological processes, pathogenic processes, pharmacologic reactions to therapeutic intervention, or any other measurable diagnostic indicator for determining the risk or presence of a disease. These signals, or biomarkers, can be detected in bodily fluids and/or tissues. Disease biomarker detection that is accurate, generally non-invasive, and easy to execute, even in point-of-care (POC) settings, can enhance disease screening, diagnosis, prognosis, and recovery. As a result, with early and prompt identification of disease biomarkers, transmission of contagious illnesses can be contained, and the mortality rates from cancer, strokes, and infectious diseases can be significantly reduced.

Researchers examined progress in biomarker identification utilizing low-cost microfluidic devices for disease diagnosis in resource-constrained settings, with a focus on infectious illnesses and cancer diagnosis. Various microfluidic platforms were used for illness diagnosis and highlighted numerous detection strategies for biomarkers. In labs, infectious diseases, cancer, and other diseases are usually identified using biomarker detection techniques such as western blotting, enzyme-linked immunosorbent assay (ELISA), immunofluorescence, immunodiffusion, polymerase chain reaction (PCR), and a variety of other techniques (Wild, 2013). However, many of these assays are sophisticated, require hours to complete, necessitate huge quantities of samples and reagents, and involve bulky and expensive devices, restricting their use in rural areas and underdeveloped countries. On the contrary, microfluidics technology has unique features for easy, low-cost, and speedy disease diagnosis, such as minimal reagent usage, quick analysis, high mobility, and built-in processing and analysis of complex biological fluids with great sensitivity. These devices provide on-chip POC diagnostics and real-time illness detection using small volumes of bodily fluids. Hence, they may serve as a link to enhance the global healthcare system's efficacy and sensitivity, particularly in rural places with limited resources, such as developing countries, home healthcare settings, and emergency scenarios. Due to all these critical properties, several microfluidic devices have been created for biomarker identification in disease diagnosis, including various forms of cancer, infectious diseases, meningitis, cardiovascular disease, and Alzheimer's. Microfluidic platforms include glass, paper-based, poly(cyclic olefin), polydimethylsiloxane (PDMS), poly(methyl-methacrylate) (PMMA), and hybrid devices. The material chosen is determined by the detection system, research application, fabrication facility, cost, and other parameters like thermal conductivity, chemical resistance, dielectric strength, and sealing qualities. Colorimetric results may be seen with the naked eye or processed using software on a computer or mobile application. “Quantity One” is a software used to measure the standard curve's intensities and examine the results. Magnetic nanoparticles bind to the target and then penetrate chambers containing ELISA reagents. The HRP substrate color change in the PMMA-based device could be photographed and analyzed using Matlab^®^ using a smartphone camera (Sanjay et al., 2015).

Additionally, DNA biosensors have been incorporated into a wide range of applications, including molecular and medical diagnostics and drug screening. They represent a more efficient method of evaluating DNA structure. They overcome the limitations of other sensors because of their quick response, accessibility, selectivity, and awareness. Electrochemical DNA biosensors, particularly, drew lots of attention due to their short communication time and high level of awareness. These sensors have been demonstrated to be effective at identifying anti-cancer chemicals, biomolecules, toxins, and neurotransmitters (e.g., epinephrine, norepinephrine, and dopamine), as well as Parkinson's. DNA biosensors can be especially helpful for medical IoT because they help diagnose genetic disorders as well as illnesses caused by changes to DNA or gene sequences (Aledhari et al., 2022). Furthermore, these types of sensors do not require expensive apparatus, making them an efficient option.

Ingestible biosensors are also thought of as an advanced variant of diagnostic wearable biosensors. They are electronic devices composed of many electronic components utilized for disease detection and monitoring. Ingestible biosensors' size is comparable to that of a capsule that humans swallow. These biosensors may address critical elements, including density, size, physical structure, and aerodynamics, to allow for facilitated digestion even when the body is in motion. Further, ingestibles are available in several forms, including imaging capsules, temperature sensor capsules, pressure sensor capsules, and others. Yet, in terms of design, ingestible biosensors must be composed of biologically compatible materials to remain functional while remaining safe from body interactions with the recipient (Ray, 2020).

Not all humans physiologically react in the same way to a certain stimulus or drug, and that is totally normal; it is due to genetic variations, mutations, environmental factors, different lifestyles, etc. Below, we emphasize this area and suggest how deducing such information could help serve patients.

### 4.4. Genetic variations and gene libraries

The rapid decline in infectious disease death rates throughout the early 20^th^ century, accompanied by a significant rise in life expectancy, was not the consequence of biological discoveries. Medical researchers looked for explanations in biological distinctions between races. When US medical and public health specialists detected that diabetes prevalence was rapidly rising in the late 19^th^ century, they also recognized that the disease did not affect all groups equally. Those who are financing the precision medicine program are at a fork in the road. They could continue investing extensively in the omics sciences, enticed by the prospect of developing personalized medicines through improvements in molecular biology and DNA sequencing. The results will undoubtedly open new markets, which explains the pharmaceutical and biotechnology industries' keen interest. The dismissal of the “one-size-fits-all” concept in medical care must be broadened to acknowledge that people are distinct not only biologically but also regarding where and how they spend their time (Tuchman, 2022). Due to this diversity, gene libraries could greatly serve patients as well as health practitioners since they help screen for target DNA fragments contributing to complex phenotypes.

After coming to the realization of genetic variation and gene libraries, we ought to make use of this information to enhance patients' treatment plans with better fits rather than using the “one-size-fits-all” approach. For so, the below sub-section will state in-depth knowledge regarding this issue.

### 4.5. Precision medicine

Personalized predictive analytics is a new approach to healthcare delivery that is based on patient similarities. When a patient requires therapy, comparable patients are discovered in archived databases, observations are derived from previous records, and individualized interpretations are conducted as per their DNA. This method is utilized in drug recommendation systems to detect risk indicators for comparable patients and provide individualized medical treatments. Precision medicine focuses on individual features such as environmental, omics (genomics, metabolomics, proteomics, etc.), phenotypic, social, and psychological factors. It is resource-intensive, patient-centered, and data-intensive. The process begins with the collection of information from sequencing the whole genome, and based on the data similarities of previous patients, BDA can thus make interpretations and aid in precision medicine (Wang and Alexander, 2020). Precision medicine could revolutionize the way healthcare is delivered, allowing clinicians to predict and prevent diseases, tailor treatments, and ultimately improve health outcomes.

Recently, the potential of precision medicine to reduce health inequities in diabetes has been investigated. Precision medicine offers the potential to correct this imbalance by harnessing BD tools to learn more about biomarkers as well as the social, physical, and environmental contexts. According to Tuchman, using BD to collect data regarding biology, lifestyle, and the environment can shatter the often-erected barriers between genes and environments (Tuchman, 2022). However, at least two issues prevent precision medicine from having a significant impact on the reduction of health inequalities. First is the cost. Regardless of where one sits on whether precision medicine will ever be obtained, it is commonly acknowledged that it will be a long time before this approach assists those who have few resources. Precision medicine may thus aggravate health inequities before it has the potential to eliminate them. The second issue is the overemphasis on omics that now characterizes precision medicine. If the purpose of precision medicine is to abandon the “one-size-fits-all” strategy to treatment, then significantly more data is needed about the radically varied material and social settings in which people live and work.

One great concern to keep at the forefront of our minds when dealing with patients is compliance. If one was asked to take 20 pills a day, for instance, they would miss or feel discouraged to follow such a prescription. Likewise, with diagnostic tests, patients opt for convenient solutions. The following sub-section discusses this matter.

### 4.6. Patient compliance

Another concern in the healthcare sector is Diabetes Mellitus. Diabetics must regularly monitor their blood glucose levels to treat their condition efficiently. A blood glucose meter is often used to test blood drops released via a finger prick with a needle. Normally, the testing frequency is determined by the diabetes type and medications taken; however, daily testing is indispensable. For diabetics, this form of testing can be challenging; fear of needles, the high cost of test strips, contamination, and the difficulty of self-monitoring are all limitations to effective blood glucose control. Fortunately, health informatics is on the verge of altering the status norms, and finger-prick testing for blood glucose tracking may soon become obsolete! Diabetes skin patches are one example of a health analytics application that opts for better patient compliance and will be discussed in Section 5 (Lipani et al., 2018).

Finally, social media insights could generate data that helps us interpret and terminate actions or even prepare for disease outbreaks, and if not traced, could threaten a lot of lives. In the last sub-section, you will learn more about social forecasting.

### 4.7. Future societal interpretations

Beheshti, Hashemi, and Wang introduced a social DA pipeline that allows analysts to interact with social data to evaluate the possibility of online radicalization (Beheshti et al., 2021). Influence maximization, or the problem of identifying a small fraction of nodes in a social network that may maximize influence propagation, has the potential to be a useful tool in identifying and predicting mental health concerns such as suicide, bullying, and radicalization. Predictive analytics in mental health, for instance, can allow for the analysis and exploration of the factors that influence people to engage in extremist activities. Assessing social influence on mental health is complicated and necessitates techniques for:

- identifying a few nodes in social networks that can increase the propagation of mental health influence, i.e., maximize influence, and
- discovering, interpreting, and communicating meaningful patterns in social data to explore the potential personality dimension.

In the next sections, we discuss and explain how these different technologies have contributed to the development of the healthcare industry and opened doors for healthcare analytics.

## 5. Healthcare analytics

In the below subsections, we study how interconnectivity between (contemporary) healthcare and DS has been reflected in several studies to address healthcare problems or provide solutions using BDA, DS, AI, ML, or IoT technologies.

### 5.1. Big data in healthcare analytics

Due to the benefits provided by BDA, its popularity has grown in various domains, such as healthcare services, medical research, and other areas (Dadkhah and Lagzian, 2019). BDA can be used to manage data-driven decisions, allowing for a more comprehensive view of medical conditions and treatments, thereby improving patient care. AI, wearables, and IoT are among the techniques employed in the healthcare sector (Wang and Alexander, 2020). For instance, industry-precise medicine uses AI, next-generation technologies, and IoT to make sense of BD. A smart healthcare architecture based on IoT technology has advanced for anyone throughout exercise; the Bayesian belief network employs an artificial neural network (ANN) model to anticipate a patient's health-related vulnerability (Awrahman et al., 2022). Moreover, data warehouse technologies are employed in the integration of healthcare data management systems to classify, segment, cluster, and analyze health data (Andreu-Perez et al., 2015).

The healthcare industry has numerous applications that utilize BDA, AI, and ML, and other areas could benefit from incorporating these technologies to improve patients' health, life expectancy, and quality of care. BDA can anticipate disease outbreaks, pharmaceutical and medical breakthroughs, individualized and precise patient care, telediagnosis, e-consultation, and more. BD in life sciences is brought about by high-throughput molecular assays, such as microarray, a subclass of such technology that presents life sciences to large datasets, allowing the study of gene expression, genetic mutations, and/or medications' effects on gene expression and cell growth (Wang and Alexander, 2020). Since AI discovers biomarkers early, BDA can also help anticipate many fatalities. In e-health, BD handles massive amounts of real-time healthcare data, identifying life-saving measures or medication discontinuance. BD can also detect sleep architecture and insomnia using clinical databases to help explain sleep medicine (based on phenotyping). The bigger the dataset, the more probable it is that the research findings or conclusions are approximate to the actual population (Awrahman et al., 2022).

In healthcare, Biomedical Big Data (MBD) has received significant attention due to the sensitivity and implicit vulnerability of health data, as well as its significant potential to enhance diagnosis, foster medical treatment, and prevent diseases (Wang and Alexander, 2020). The digitalization of healthcare data is the product of BD innovation and revolt. Healthcare data comes in a variety of formats, including biological signals, genomic and sensor data, biomedical imagery, and social media. Genomic data informs people about genetic markers, consanguinity, disease conditions, and mutations; clinical text-mining converts data from unorganized practical medical notes to suitable information via information extraction and natural language processing (NLP), which draws useful data from a massive volume of text (Verma and Sood, 2018).

Data management, processing, and retrieval are vital in healthcare. For an efficient data discovery process, the right data should be collected at the appropriate time and in the appropriate context. For context-awareness in healthcare applications, the split between various professions, such as medical science and computer science, must be bridged. The gap between structured and unstructured data has been bridged by BDA, yet the shift to an incorporated data environment is a well-known barrier to bypass. However, in a short period, we saw a variety of analytics in use with positive effects on healthcare sector decisions. When integrated with organized and unstructured EHR data, predictive analysis may be more efficient. Clinical events can be retrieved from EHR data, and similar sentences can be classified semantically. Distinct and scattered representations can successfully forecast clinical results when using semantic spaces to extract clinical language from the EHR. Health records can be used to collect many types of information for objectives such as phenotyping, pharmacovigilance, and sickness detection. Social media analytics aid in the comprehension of society's most prevalent ailments, in addition to psychological disorders. When compared to other fields, social media analytics has the greatest difficulties because reviews, postings, and comments cannot be standardized. Several linguistic difficulties obstruct clean analytics. However, database aggregation and data cleansing, as part of effective healthcare analytics, may lower data heterogeneity, lack of organization, and other quality issues (Awrahman et al., 2022).

### 5.2. Data science in healthcare analytics

One of the examples of DS use in healthcare is disease analytics. Disease analytics, healthcare analytics, and/or medical analytics serve to reveal disease-related traits and provide people with a better understanding of diseases. As a result, it aids in the prevention and treatment of the condition. Generally, disease refers to disorders that cause discomfort, dysfunction, distress, or death in infected people and their close contacts (Leung et al., 2021). Additionally, the development of a social DA pipeline to enable analysts to investigate suspected online radicalization using social data is another example of the utility of DS in healthcare. Novel cognitive graph, entity, and connection concepts are introduced to model and understand elements that drive extreme and criminal conduct. The concept of a “knowledge lake” has been established to offer the foundation for BDA by automatically curating raw social data and processing it to derive insights. They planned to expand the knowledge lake by enriching social things (e.g., a tweet on Twitter) with features relating to social actor behavior (Beheshti et al., 2021).

HCloud is an example of cloud computing services for medical applications that include physiological signal data analysis and illness early warning systems. Cloud computing applications for the medical field are concerned with the storage, access, and management of confidential health data rather than utilizing the complete computational capacity of cloud platforms. Real-time embedded signal processing might be built into chips implanted in mobile phones or clothing for patients to monitor remotely. The huge amount of data obtained from biological sensors contains personal data, which mandates data privacy protection by design. The modeling of cloud computing architecture is handled without privacy compromising. By granting user credentials to remotely login to the cloud server and dynamically verify the status of the cloud services available, data encryption is necessary for security and data protection. While shared infrastructure and the WPN (wireless personal area network) are employed, patient data must be housed in a private cloud. In the case of electrocardiogram (ECG) monitoring, for example, cloud computing technologies enable remote monitoring of a patient's cardiac data, rapid data interpretation, and warning of first-aid staff and doctors if data shows potentially serious conditions. The use of cloud computing technologies in healthcare reduces costs associated with patient monitoring in hospitals. The benefits of cloud computing in healthcare include storage spaces for BD from biomedical sensors; data security and privacy by design; data availability; DA and predictive modeling; data classification and analytics; decision support in medical acts; and cost savings for hospitals and healthcare providers (Aileni et al., 2015).

### 5.3. Artificial intelligence and machine learning in healthcare analytics

AI and ML have been deemed deserving of identifying biomarkers (Rescinito et al., 2023). AI, due to its accuracy and fast diagnostic speed, has been used in dermatology, pathology, and radiology for image analysis, resulting in decreased medical errors, the recommendation of precision therapies for complex diseases, optimization of chronic illness care procedures, and increased patient enrollment in clinical trials- all of which are benefits of automation. Additionally, algorithms examine patterns in massive amounts of data to interpret trends and outbreaks.

The use of Neural Networks (NNs) to assess disease data is a popular strategy. NNs are groups of connected nodes (i.e., artificial neurons) that loosely imitate the neurons in a biological human brain. These nodes add their inputs and send weighted sums (e.g., numbers) to other nodes as signals. Further, these nodes are typically compiled into layers, with signals traveling from the input to the output layer via numerous intermediate levels. For training, these NNs typically require a large amount of data. Existing efforts on sensing, tracking, testing, diagnosis, therapy, and prognosis include, for example, the imaging-based diagnosis of COVID-19, which employs many Chest-computed Tomography (CT) scan pictures for training. However, these images can be expensive to develop, and the technology used to create them (e.g., CT scanners) may not be commonly available (Leung et al., 2021).

**Figure 4:**
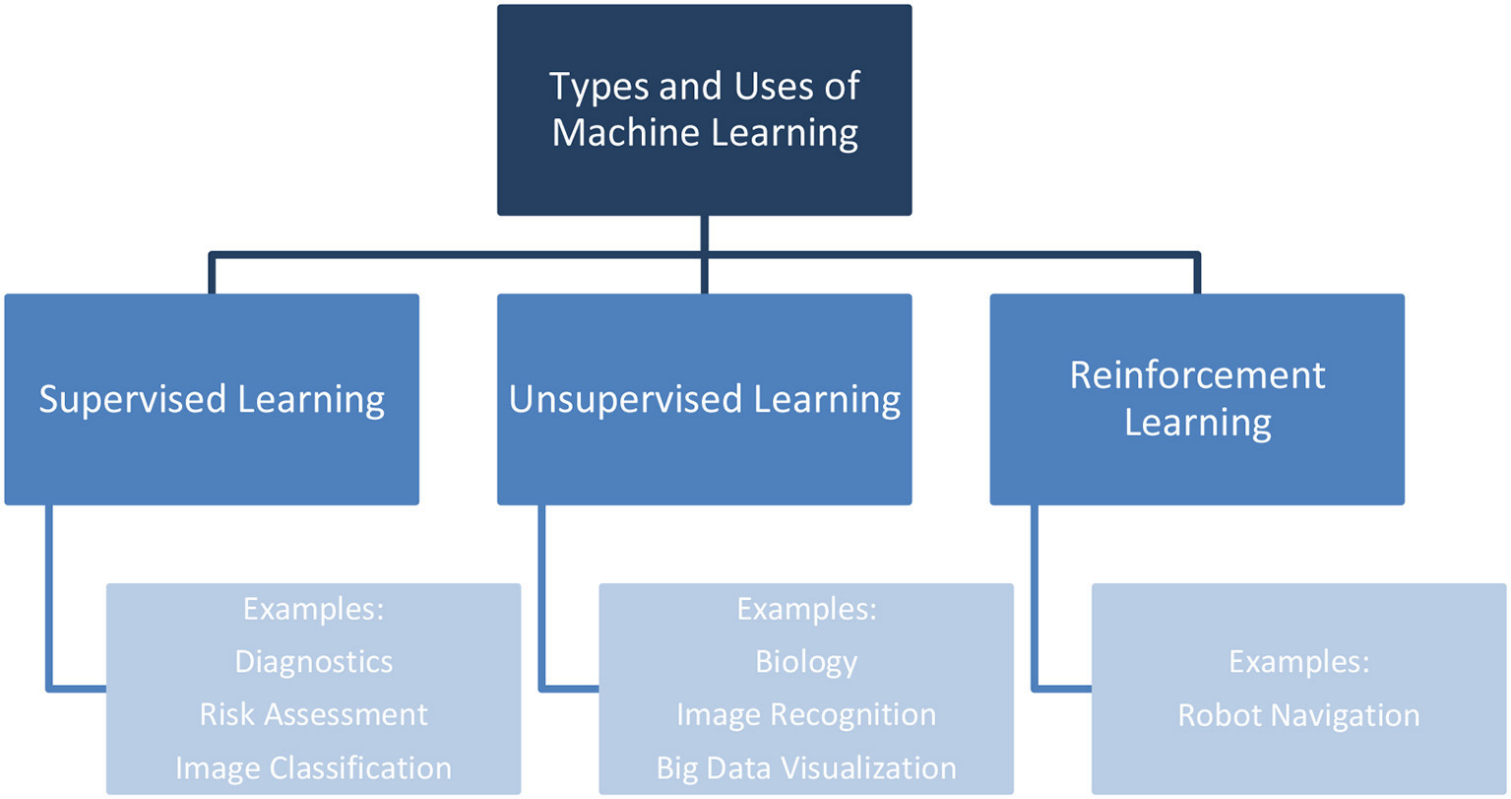
Types and applications of ML in healthcare.

Intelligent agents, ML, and text mining are three technical (3T) branches that have contributed to healthcare. ML is a branch of AI that uses data and algorithms to mimic how people learn, gradually increasing its precision. ML approaches are being used in a variety of scientific domains, resulting in more evidence-based DM (Meszaros et al., 2022). A broad distinction is made between ML algorithms based on whether they employ labeled or unlabeled training data and whether the goal is to anticipate certain outcomes or to find patterns in the data. Unsupervised, supervised, and reinforcement learning are the three main types of ML. Supervised learning trains on labeled data, builds a model, and then classifies novel observations. Unsupervised learning identifies hidden patterns in unlabeled data. Finally, reinforcement learning employs a feedback mechanism to maximize cumulative reward and enhance outcomes (Wang and Alexander, 2020; Awrahman et al., 2022). Figure 4 represents the main types of ML along with some applications in the healthcare industry.

Deep learning (DL) is a group of ML that employs layered computational nodes, with each node in each layer doing computations on inputs and their associated weights. It is commonly utilized in speech analysis, object recognition, and object detection (Shoaib et al., 2023). These techniques are increasing in popularity because they have the capacity to perform at the human level in a variety of medical disciplines, such as cancer detection, diabetic retinopathy detection, neural connectomics, molecular drug activity, genomics, speech recognition, image recognition, disease modeling, and risk prediction. It is critical to comprehend how DL can save lives (Harerimana et al., 2019; Witt et al., 2019; Ali et al., 2021; Meszaros et al., 2022). DL is more successful than other ML algorithms since there is no need to invest additional time and effort in feature building with a domain expert; instead, the system can learn the features from raw data, classify the images, and localize tumors/diseases. However, due to the complexity of EHR data and the special intolerance to errors, feature depiction and selection, which are typically guided by a domain expert, may be critical to the success of a DL model (Harerimana et al., 2019).

ML approaches based on AI and historical databases have been utilized to manage massive data volumes. Based on contemporary biomedical data concerns, ML algorithms play an important role in managing MBD (Wang and Alexander, 2020). Various ML algorithms for forecasting health conditions have become popular subjects of interest in academia, industry, and healthcare research. Traditionally, the goal of medical research is to use ML algorithms with clinical data (e.g., age, gender, symptoms, vital signs, physical examination findings, lab values, imaging variables) to foresee clinical outcomes or identify relationships between predictor variables and clinical outcomes. Although wearable data can be paired with larger sets of patient data to add context, raw sensor data can also be utilized as direct input into ML algorithms to predict a clinical result (i.e., physiologic or pathophysiologic states) or identify meaningful data aspects (Witt et al., 2019).

### 5.4. IoT in healthcare analytics

The aging population, scarcity of healthcare supplies, and rising medical expenditures require the development of IoT-based technology to solve these difficulties in healthcare. The most significant impacts of IoT in healthcare entailed improved information exchange, shorter hospital stays, and lower healthcare expenses. IoT applications include m-health, wearable gadgets, online monitoring of patients, and indirect emergency healthcare, particularly at home (Dadkhah and Lagzian, 2019). The widespread adoption of IoT technologies has enabled organizations to enhance work processes and increase productivity by collecting and reporting environmental data. These items can also respond autonomously to happenings in their surroundings. All these processes can be activated to perform actions and provide services through human intervention or M2M communication. Remote patient monitoring has enormous potential for improving healthcare quality as well as lowering expenditures by detecting and avoiding diseases and dangerous situations. Although IoT has been around for more than a decade, two discoveries have been the key motivations driving its expansion. First is the rapid expansion of mobiles and applications, and second is the widespread availability of wireless access (Ahmadi et al., 2019).

Even though healthcare is one of the domains that greatly benefits from IoT, most researchers in this domain are technical personnel rather than health practitioners and experts. Moreover, most articles on IoT for healthcare are published in technological journals or conferences, highlighting the major issue of inadequate exposure of healthcare staff to this sector. One way healthcare professionals can help medical-based IoT research is by taking into consideration the technical aspects (frameworks, platforms, infrastructures, communication technologies, building IoT-based systems, etc.) of IoT usage in medical applications. Second, academics could help promote medical-based IoT research by managing the technology in the healthcare sector, a process known as “IoT management”. Healthcare professionals could also participate in the testing process, where they would be able to use a developed system and provide input on its usability. Their involvement extends beyond the requirements, operation, and testing phases. Instead, they can be engaged from the very early stage to provide developers with insights into new IoT applications in specific disciplines or discover new ones for which IoT is appropriate. These innovative applications could either improve the current system or pave the way for new improvements. All the aforementioned inputs from health experts will aid in the advancement of IoT research in the healthcare arena (Dadkhah and Lagzian, 2019).

Wearable devices (aka wearables), including sensors, are increasingly incorporated into a range of commercial devices utilized to gather and process raw physiological indicators into meaningful digital health information. Wearable data is frequently referred to as “big data,” which brings both opportunities and drawbacks. Wearable data is now studied across a wide range of therapeutic areas and patient populations. Many consumers' wearables capture physiologic data such as skin temperature, heart rate (HR), and peripheral capillary oxygen saturation, as well as data on geolocation, physical activity, and other environmental factors. The National Health and Nutrition Examination Survey studies and the UK Biobank Study cohorts were early population-level study initiatives that gathered physical activity data via wearables (Witt et al., 2019).

Before undertaking any more advanced analytical tasks, the data collected by the various sensors in many types of wearables must often be preprocessed. These issues are commonly categorized as signal processing and other possible data preprocessing phases such as filtering, labeling, segmentation, and feature extraction and selection. In a discussion of wearable biosensing, Celka et al. defined an architecture for data fusion that includes collecting information from several biosensors and transferring the data through a low-level sensing layer (e.g., local sensors, signal processing, and local signal conditioning), then through a higher-level processing layer divided into two global sublayers: feature extraction/selection and classification (Celka et al., 2005).

Moreover, while the peri-procedural chance of symptomatic stroke is modest, multiple investigations have found a substantial number of silent ischemic brain lesions, most of which occur in locations with little clinical significance. The silent brain injury may result in neuropsychological deficiencies or, worse (e.g., pre-existing dementia) implying the necessity for a rigorous evaluation of the influence of these treatments on neurological function. According to some data, cerebral embolism is underreported, and the rate of silent ischemic stroke or silent cerebral ischemia is substantially greater than reported in available research. Considering the lack of clinical signs, this cerebral ischemia's cumulative effect could be connected to dementia and cognitive dysfunction. In light of these factors, significant emphasis ought to be placed on cerebral embolism and preventive methods to limit the risk of silent cerebral ischemia during and after surgery (Ciccarelli et al., 2022). This could be achieved with the aid of BD and AI, where high-risk patients get to be supplied with wearable devices by their healthcare provider. These wearables will detect biomarkers and be linked to cloud-based storage, where data from the patient is uploaded in real-time and hence alerts caregivers for early signs so that constructive or preventive measures, or even early admission to the hospital, can be taken to minimize complications and deaths.

Another IoT application that researchers should earn praise for is developing an adhesive skin patch that checks glucose levels at 10-to-15-minute intervals for diabetics. This innovative skin patch proved to be a feasible and non-invasive approach for monitoring blood glucose levels in skin. The skin patch has microscopic sensors that utilize electric currents to pull glucose from the interstitial fluid in follicular channels using electroosmotic extraction. The patch stores glucose in little “reservoirs,” which are monitored every 10 to 15 minutes. The goal of the patch is to send glucose readings to patients' smart phones/watches and inform them when medication is needed. The patch is not designated to puncture the skin, which is crucial because it eliminates the inconvenience of needle pain, contamination, and so on. Furthermore, because it can measure glucose from such a tiny region on hair follicles, it is extremely precise, eradicating the need for blood collection to corroborate the findings. The researchers still aim to improve the accuracy of the glucose skin monitoring patches. The team plans to prolong the glucose monitoring time to 24 h and increase the number of sensors it has in the future. This could, hence, improve the patients' compliance, along with all the afore-mentioned pros (Lipani et al., 2018). An IoT application in ophthalmology was also presented by Prouski, Jafari, and Zarrabi. The eyeglasses in this model transmit signals from the eye blood flow sensor and the lens color to display eye bleeding (Prouski et al., 2017).

ACTIVAGE is a large-scale European multi-center pilot project on smart living environments. The main goal is to build the first European IoT ecosystem across nine Deployment Sites in seven European countries, reusing and scaling up underlying open and proprietary IoT platforms, technologies, and standards, and integrating new interfaces required to provide interoperability across these heterogeneous platforms, enabling the deployment and operation at large scale of Active and Healthy Aging IoT-based solutions and services, supporting and extending older individuals' independence in their living settings, and responding to the demands of caregivers, service providers, and public authorities. ACTIVAGE enhances the health, independence, and quality of life (QoL) of senior people, due to the cutting edge of technology; like IoT. It is a large-scale pilot in the field of smart living environments, supported and financed by the EU. Aging is a global physiological phenomenon, ACTIVAGE aims for enhancing services in late life for better healthcare. The goal was to create an ecosystem for active aging solutions based on IoT technology to support independent living of elderly people and promote QoL across Europe, for better use of time, leisure, and provide tranquility for both the elderly and the care givers. IoT helps care givers/families know where the person is located, what they are/are not doing, and if they are good/bad. Chronic and serious issues need to have coordinated and integrated care and for doing so technology is needed. The impact on the QoL is the impact of studies made by researchers for ACTIVAGE. IoT are devices everywhere, we take them with us, wear them, they are all around us. These devices can sense the environment around us and are even capable of changing these environments (ACTIVAGE Project, 2019).

To sum up, wearable physiological sensors collect real-time biological signals from patients such as temperature, pulse oximetry, oxygen saturation, blood pressure, respiratory rate, ECG, and so on. A cloud-computing system synchronizes this data with installable apps for analysis and storage, allowing patients to submit their indications and symptoms to the caregiver (Awrahman et al., 2022). BDA and IoT are projected to be critical technologies to assist the future generation of eHealth and mHealth. This brings us to the realization of the importance of implementing such tools for outpatients!

This reflects that the rise of IoT allows for a more personalized approach to providing healthcare for the next generation of m-health solutions. In context, this technology has the potential to define not only novel patient and physician communication options but also more targeted therapeutic strategies for patients. In e-health, medical devices are linked to the internet to provide telehealth services (e.g., external/internal monitoring and teleconsultations) (Uhm et al., 2017; Zaman et al., 2023). Uhm et al. identified in their study the credibility and applicability of m-health tools for breast cancer and some chronic diseases, including obesity and diabetes (Uhm et al., 2017). Further, mobile phone applications can feature a variety of instructional materials, allowing users to manage their calorie intake and physical status as well as interact with information and support providers. Overall, these factors result in lifestyle modifications and improved health outcomes.

## 6. Research gaps

Our paper has reviewed the literature pertaining to the use of BD and analytics in healthcare. The literature review revealed gaps that require further investigation. We outlined those gaps in this section.

As a result of the benefits brought by IoT, its adoption has grown in various fields (Dadkhah and Lagzian, 2019). Howbeit, despite its bright potential, the emergence of AI and automated decision-making (ADM) in healthcare services and research is beset by technological, regulatory, and ethical hurdles. The primary difficulties are the absence of compatibility and standardization within medical IT systems. Building trust in care interactions requires reliability and transparency, and the opacity of AI applications may jeopardize such ties. Furthermore, in unique occurrences of pharmacological adverse effects and underrepresented populations, algorithms may underperform, potentially leading to discrimination. Technology offers untapped prospects for the further utilization of health data in the age of BD and AI. To get clear consent from a considerable number of data users for new processing objectives, disproportionate efforts would be required, posing legal, ethical, and technical hurdles. As a result, academics and politicians are increasingly challenging the purpose limitation principle to deliver more efficient treatment while conserving money.

The General Data Protection Regulation's (GDPR) rules on profiling and “solely” ADM have had a substantial influence on the use of AI in healthcare and research. It is critical to distinguish between profiling, ADM, and solely ADM. Profiling is defined by the GDPR “any form of automated processing of personal data consisting of the use of personal data to evaluate certain personal aspects relating to a natural person, in particular to analyze or predict aspects concerning that natural person's performance at work, economic situation, health, personal preferences, interests, reliability, behavior, location, or movements” [Shoaib et al., 2023, p. 3]. Profiling's primary significant features are the automated processing and analysis of a natural person's personal qualities. It may entail assessing or judging a person, as “evaluation” implies. Profiling does not imply a simple classification of individuals. Profiling is not considered when a healthcare professional selects patients based on age or gender without making assumptions or completing additional assessments (Meszaros et al., 2022).

ADM refers to an automated decision made about a person with human engagement, while “solely ADM” refers to a choice made purely by an algorithm with no meaningful human involvement. Profiling, on the other hand, can be a source for both sorts of ADM. In this aspect, “solely ADM” has a greater impact on healthcare services than research since the fundamental purpose of scientific research is to develop novel expertise rather than make opinions regarding individuals. The GDPR allows data controllers to use profiling and ADM based on lawful grounds with sufficient safeguards. Howbeit, relying purely on ADM is prohibited, with certain exemptions such as explicit permission or Member State law (Meszaros and Ho, 2018).

One major and crucial aspect that must be taken into consideration is that building and training AI systems necessitates massive amounts of precise data, some of which may include private medical data in medical services and research. As a result, under the GDPR, data privacy is a key legal issue, particularly in the EU. Health data has traditionally been gathered and handled for specific reasons, like diagnosis and direct care. Therefore, the purpose limitation concept is incorporated into data protection and medical regulations around the world, which indicates that health data should not be analyzed for another objective unless specific criteria are met. Health data are identified by the GDPR as “personal data related to the physical or mental health of a natural person, including the provision of healthcare services, which reveal information about his or her health status.” The GDPR usually forbids the processing of sensitive data, like health information. However, it gives various exceptions to this rule, including public health crises like the COVID-19 pandemic (Meszaros et al., 2022).

BD posits several concerns that commonly occur in healthcare businesses since MBD contains a large quantity of unstructured data, such as handwritten documents. Clinical BDA requires acquisition, storage, integration, and visualization, which jointly present a reasonable level of difficulty (Dimitrov, 2016; Sarkar, 2017). It is ineffective for organizations to share structured data, yet exchanging unstructured data is also challenging. Therefore, it is a significant problem to successfully mine massive amounts of unstructured data. However, there are challenges associated with data storage, search, capture, sharing, analysis, data quality, real-time processing, privacy, security, and heterogeneous data too (Awrahman et al., 2022).

Additionally, one of the most challenging tasks when dealing with massive amounts of data is security. To have a significant impact, BD needs to access practically everything, including social media lives and private recordings. However, the cost of disclosing private information has been paid. Moreover, there is no patient autonomy. There are rules in place to protect the privacy of medical recordings; they are not considered, however, because it is felt that someone's knowledge should not be prohibited when it concerns their health (Abouelmehdi et al., 2018). Further, the problem of rapidly expanding noise data is crucial; varied outcomes are created by varying levels of quality and completeness, resulting in misleading findings (Sacristan and Dilla, 2015).

For BDA, accuracy is vital; personal health records (PHR) might include typing mistakes, abbreviations, and random notes; medical personal data input may have mistakes or may be put in a false environment, affecting the efficiency of gathered data instead of being submitted in a clinical setting by a professional trainee or medical practitioner; and gathered data from social media may end up in false assumptions (Andreu-Perez et al., 2015). Also, finding adequate labeled data for training is one of the most significant barriers to applying ML to EHR data. For example, if we are studying CT scans to detect a dangerous tumor, we might not be able to find sufficient recorded events to train a model (Harerimana et al., 2019).

Lastly, there has been research on generic frameworks and illness-specific studies, like diabetes, cardiovascular disorders, cancer detection, Parkinson's, and Alzheimer's diseases. Monitoring devices, such as video-imaging, robotic microscopy, databases, and IT applications, could subsequently be used as tools for large-scale research and therapy customization. Hence, when best-effort internet connections are inadequate for some applications (e.g., to mimic the impact of a microscope), the existence of restricted virtual pathways to fog services at the edge may help bridge the gap (Awrahman et al., 2022).

## 7. Research agenda

The literature synthesis provided by our literature review and the gaps identified pave the way for future research in healthcare analytics. In this section, we present a set of research questions that we propose to address in future research. Toward that end, since the below-listed research questions are neither inclusive nor prescriptive, we expected researchers to use them, in part or in full, as a base to conduct further research of different types (i.e., quantitative, qualitative, experimental, etc.) in the area.

According to the reviewed literature, data privacy and security are important points of discussion that deserve further research. Examples of these issues are data leakage and secondary use of data, data ownership, and cyber-attacks. Examples of questions that can be explored are:

- *What data privacy and security measures for wearable physiological sensors should be taken into consideration?*
- *How may data privacy and security concerns affect analytics algorithms in the healthcare domain?*
- *What risks may cloud computing create for users in terms of data privacy and security? How to overcome these risks using DA?*

Besides privacy and security concerns, some studies have highlighted other ethical aspects of using DA in the healthcare sector, such as human-centered AI solutions. Therefore, the question could be:

- *How should AI solutions be designed and developed to meet human needs while avoiding the dehumanization of smart healthcare solutions?*

Many of the reviewed articles have highlighted the importance of the adoption of healthcare solutions by both individual users and organizations when healthcare benefits from BDA. According to the literature, far more work needs to be done to understand the adoption barriers of smart healthcare tools and services. These questions can, for example, be:

- *How BDA may contribute to building trust for healthcare users to increase the adoption of smart healthcare solutions (e.g., use of IoT sensors)?*
- *In what ways may BDA increase or decrease the adoption rate of smart healthcare technologies such as telemedicine?*

Several studies have touched upon the effects of BDA on healthcare organizations and healthcare systems. However, these studies have not specifically investigated how and in what ways healthcare systems might be affected in the BD era. Therefore, the following RQs can be good directions for future work:

- *How and in what ways may BDA transform the management and organization of the healthcare system in the future?*

Many of the reviewed articles have discussed the impacts of BDA techniques and associated tools such as IoT on QoL. However, these discussions remained inconclusive. One crucial question to investigate is:

- *How may using BDA in healthcare affect humans' QoL in terms of both benefits and threats?*

There have also been discussions in the reviewed articles regarding environmental sustainability in the healthcare sector when BDA comes into play. However, these articles have not thoroughly investigated this aspect, and further research is required in this regard. For example,

- *In what ways may using cloud computing in healthcare affect environmental sustainability?*

The cross-disciplinary nature of healthcare analytics relies on its roots in data and analytics algorithms; therefore, addressing the below is vital:

- *How to design the appropriate data architecture to facilitate healthcare analytics for healthcare organizations and medical staff?*
- *How to select the appropriate algorithms for analytics? How to configure them?*
- *How can we address the knowledge shortage that medical staff and organizations currently face and benefit more from prediction for the benefit of patients and human health?*

Furthermore, in data-driven healthcare decisions or automated decisions, we propose the following questions:

- *How do we ensure the transparency and explainability of the recommendations made by algorithms?*
- *How to avoid addressing the issues of aversion, accountability, and complacency while healthcare staff use algorithms?*

## 8. Conclusion

This research represents a response to the collective call by researchers for investigating “how” DS may help the healthcare sector address and solve current challenges (Dadkhah and Lagzian, 2019; Awrahman et al., 2022). Most research in the field has largely discussed “why” DS is important to be used to overcome healthcare challenges. However, the question of “how” they be tackled has not been sufficiently explored. In doing so, this study contributes to the body of knowledge by providing an in-depth analysis of how contemporary technologies such as DS, BD, AI, ML, and IoT have been used within the healthcare area.

A literature review, such as this study, constructs a well-founded foundation for advancing knowledge in the area of investigation. It enables theory development and helps uncover research areas where further efforts are desirable. A literature review study aims to reveal the sources relevant to an area under investigation by a particular study. It, therefore, constructs a vital contribution to the relevance and rigor of research. Relevance is improved by circumventing the reinvestigation of the known. Rigor is originated from relying on the underlying knowledge base. The value of literature review studies is unquestionable (Webster and Watson, 2002).

Regarding the proposed research agenda, by identifying research gaps, the study suggests research questions that could be explored and addressed by researchers, practitioners, and government healthcare agencies and officials, pertaining to the technical challenges of articulating the data architecture, the explainability and transparency of algorithms, the compelling issues of privacy and security, and the legislative barriers. To the best of our knowledge, none of the available literature has explored the integration of DS in the healthcare sector and provided multiple directions for future research and practitioners. For instance, regarding the barriers to the adoption of DS solutions in the healthcare industry, most studies have emphasized these barriers, encompassing technical, societal, cultural, and legislative aspects. While examining how these adoption barriers must be tackled in an efficient way, this has not been explored sufficiently. According to the reviewed literature, these barriers may include but are not limited to the legislative barriers, i.e., the legal and regulatory challenges that may hinder the implementation and utilization of DS technologies and data-driven solutions in healthcare. Those legislative barriers can be related to, for example, data privacy and security regulations (Aileni et al., 2015; Abouelmehdi et al., 2018; Harerimana et al., 2019; Meszaros et al., 2022), ethics of healthcare and consent (Ahmadi et al., 2019), data ownership (Wang and Alexander, 2020), interoperability standards (Sarkar, 2017; Wang and Alexander, 2020), and licensing and intellectual property (Ahmadi et al., 2019). To overcome legislative barriers, effective collaboration among healthcare organizations, legal experts, policymakers, and technology providers is crucial. It is imperative to approach these obstacles with careful consideration, ensuring that DS initiatives in healthcare adhere to all relevant laws and regulations to safeguard patient privacy and safety. To conclude, our hope is that providing a comprehensive review could enable those interested in healthcare research to know the state-of-the-art in the area and be able to advance it further.

## Author contributions

RE: Writing—original draft, Writing—review and editing. AE: Supervision, Writing—original draft, Writing—review and editing. AH: Methodology, Writing—original draft, Writing—review and editing.
